# Behavioral Traits Associated With Resilience to the Effects of Repeated Social Defeat on Cocaine-Induced Conditioned Place Preference in Mice

**DOI:** 10.3389/fnbeh.2019.00278

**Published:** 2020-01-09

**Authors:** Claudia Calpe-López, Maria Pilar García-Pardo, Maria Angeles Martínez-Caballero, Alejandra Santos-Ortíz, Maria Asunción Aguilar

**Affiliations:** ^1^Neurobehavioural Mechanisms and Endophenotypes of Addictive Behavior Research Unit, Department of Psychobiology, University of Valencia, Valencia, Spain; ^2^Department of Psychology and Sociology, Faculty of Social Sciences, University of Zaragoza, Teruel, Spain

**Keywords:** resilience, social defeat stress, cocaine, mice, conditioned place preference, reward, vulnerability

## Abstract

The relationship between stress and drug use is well demonstrated. Stress-induced by repeated social defeat (RSD) enhances the conditioned place preference (CPP) induced by cocaine in mice. The phenomenon of resilience understood as the ability of subjects to overcome the negative effects of stress is the focus of increasing interest. Our aim is to characterize the behavior of resilient animals with respect to the effects of RSD on the CPP induced by cocaine. To this end, 25 male C57BL/6 mice were exposed to stress by RSD during late adolescence, while other 15 male mice did not undergo stress (controls). On the 2 days following the last defeat, all the animals carried out the elevated plus maze (EPM) and Hole Board, Social Interaction, Tail Suspension and Splash tests. Three weeks later, all the animals performed the CPP paradigm with a low dose of cocaine (1 mg/kg). Exposure to RSD decreased all measurements related to the open arms of the EPM. It also reduced social interaction, immobility in the tail suspension test (TST) and grooming in the splash test. RSD exposure also increased the sensitivity of the mice to the rewarding effects of cocaine, since only defeated animals acquired CPP. Several behavioral traits were related to resilience to the potentiating effect of RSD on cocaine CPP. Mice that showed less submission during defeat episodes, a lower percentage of time in the open arms of the EPM, low novelty-seeking, high social interaction, greater immobility in the TST and a higher frequency of grooming were those that were resilient to the long-term effects of social defeat on cocaine reward since they behaved like controls and did not develop CPP. These results suggest that the behavioral profile of resilient defeated mice is characterized by an active coping response during episodes of defeat, a greater concern for potential dangers, less reactivity in a situation of inevitable moderate stress and fewer depressive-like symptoms after stress. Determining the neurobehavioral substrates of resilience is the first step towards developing behavioral or pharmacological interventions that increase resilience in individuals at a high risk of suffering from stress.

## Introduction

According to the World Health Organization, the global prevalence of cocaine use was estimated at roughly 0.4% of the global population aged 15–64 in 2016 (about 18.2 million users), with higher incidence rates in developed societies (World Drug Report, 2018). Individual and environmental variables act as risk factors, facilitating the initiation and maintenance of drug use, the transition to addiction, and relapse after detoxification (Dellu et al., 1996; Enoch, 2006). Among the environmental factors affecting vulnerability to drug addiction, exposure to stress plays a primary role. Traumatic life events during critical periods of development have a profound influence on the development of personality (Kim et al., 2009; Congdon et al., 2012; Oshri et al., 2013) and increase the risk of suffering from mental and drug-use disorders (Kessler et al., 2010; Sayed et al., 2015).

Chronic social stress, including problems with social interaction (family or friend relationships, work-place stress, bullying, etc.) is the most frequent type of stress faced by human beings. In preclinical studies with rodents, chronic social stress is modeled by the repeated social defeat (RSD) paradigm. Brief episodes of aggression from a more aggressive conspecific, together with social subordination, induce anxiety- and depression-like symptoms (Bartolomucci et al., 2009; Nestler and Hyman, 2010; Hollis and Kabbaj, 2014; Czéh et al., 2016; Vannan et al., 2018). Exposure to RSD has also been shown to increase the rewarding effects of drugs of abuse (Ellenbroek et al., 2005; Burke et al., 2011; Aguilar et al., 2013; García-Pardo et al., 2015, 2017; Newman et al., 2018). Moreover, several studies performed in our laboratory using the conditioned place preference (CPP) paradigm have demonstrated that mice exposed to RSD during late adolescence exhibit an enhanced sensitivity to the rewarding effects of low doses of cocaine in adulthood (Montagud-Romero et al., 2016a,b; Rodríguez-Arias et al., 2017; García-Pardo et al., 2019).

In spite of the close relationship between life adversity and psychopathology, not all individuals exposed to stress develop a mental disorder. In fact, most are resilient and display an adaptive response to stress that ensures a relatively normal physical and psychological function (Southwick and Charney, 2012). Thus, resilience can be defined as “the process of adapting well in the face of adversity” (Charney, 2004), or as the capacity to overcome the deleterious consequences of stress, which result in the development of psychiatric disorders in more vulnerable individuals. It is unclear why some individuals are more resilient to the impairing effects of stress than others, but neurochemical, genetic, and epigenetic processes seem to be associated with resilience to stress-related disorders (Cadet, 2016; Osório et al., 2016).

The RSD paradigm has proven to be a useful model for studying the mechanisms involved in susceptibility or resilience to the negative consequences of social stress (Nestler and Hyman, 2010). As in humans, individual differences exist in the development of psychopathology after RSD exposure. Only the subgroup of mice characterized as susceptible to the effects of RSD on social interaction with a conspecific (social avoidance) exhibit a wide variety of deleterious consequences, including anhedonia- and anxiety-like symptoms, elevated reactivity of the hypothalamic-pituitary-adrenal (HPA) axis and other behavioral and physiological alterations (Berton et al., 2006; Krishnan et al., 2007; Nestler and Hyman, 2010; Russo et al., 2012; Russo and Nestler, 2013).

Resilience could also explain why not all individuals who undergo stressful experiences become addicted to drugs of abuse. Using the RSD model, Krishnan et al. (2007) demonstrated that only mice characterized as susceptible (mice that displayed social avoidance after RSD exposure) developed cocaine-induced CPP. Similarly, animals vulnerable to the effects of RSD on social interaction were shown to increase alcohol self-administration in comparison to non-stressed controls or resilient animals that did not develop social avoidance after RSD (Nelson et al., 2018). Both studies suggest that resilient mice that do not display a deficit of social interaction after stress are also resilient to the rewarding effects of drugs of abuse. These are the only studies to have identified animals that were susceptible or resilient to the influence of RSD on the rewarding effects of drugs of abuse. As Cadet (2016) noted, most neuroscience research has focused on identifying negative or pathological elements underlying a subject’s vulnerability to drug addiction; however, the characterization of the traits that confer resilience against the consequences of social stress on the effects of drugs of abuse could be a more effective approach to preventing and treating addictive disorders. Identifying predictive behavioral patterns of resilience is the first step towards developing early, individualized preventive strategies that enhance resilience and promote a resilient personality in individuals at risk who are exposed to significative levels of stress.

Thus, the aim of this work was to determine the existence of individual differences in response to RSD and to characterize the behavioral profile of animals that are resilient to the long-term effects of social defeat on cocaine-induced CPP. For this purpose, a group of late adolescent mice were exposed to RSD (four episodes separated by intervals of 72 h), while another group did not undergo stress. The behavior of the defeated mice was evaluated during the first and fourth episodes of defeat and they were segregated in two subgroups according to the time they spent engaged in defense/submission. The short-term effects of RSD were evaluated to compare the behavior of defeated mice to that of control mice in the elevated plus-maze and the hole board and in social interaction, tail suspension and splash tests, 24–48 h after the last episode of defeat. According to the behavior of the defeated mice in these behavioral tests, they were segregated into two subgroups: one affected by RSD (vulnerable mice), and the other behaving like the control group (resilient mice). Three weeks after the last episode of defeat, acquisition of CPP after conditioning with a low dose of cocaine was evaluated in all the mice in order to identify the behavioral traits that confer resilience to the long-term effects of RSD on the CPP induced by cocaine. A lack of CPP was used to define the animals that were resilient to the effects of RSD on cocaine reward since non-stressed mice did not develop CPP with the dose of cocaine employed.

## Materials and Methods

### Subjects

Forty male mice of the C57BL/6 strain and 15 male mice of the OF1 strain (Charles River, France) were used in the study. They arrived in the laboratory on a postnatal day (PND) 21 and were housed for 26 days before initiation of the experimental procedures. Experimental mice (C57BL/6) were housed in groups of four in plastic cages (25 × 25 × 14.5 cm). Mice used as aggressive opponents (OF1) were individually housed in plastic cages (23 × 32 × 20 cm) in order to induce heightened aggression (Rodríguez-Arias et al., 1998). To reduce their stress levels in response to experimental manipulations, grouped mice were handled for 5 min per day on each of the 3 days prior to initiation of the experimental procedures. All mice were housed under the following conditions: constant temperature; a reversed light schedule (white lights on 19:30–07:30); and food and water available *ad libitum*, except during behavioral tests. Procedures involving mice and their care were conducted according to national, regional and local laws and regulations, which are in compliance with the Directive 2010/63/EU. The protocol was approved by the Ethics Committee in Experimental Research (Experimentation and Animal Welfare) of the University of Valencia (A1507028485045).

### Drugs

Animals were injected intraperitoneally with 1 mg/kg of cocaine (Alcaliber Laboratory, Madrid, Spain) or (NaCl 0.9%) in a volume of 0.01 ml/g of weight. The physiological saline was also used to dissolve the cocaine. The dose of cocaine was selected on the basis of previous studies (Rodríguez-Arias et al., 2017; García-Pardo et al., 2019).

### Experimental Design

**Figure 1:**
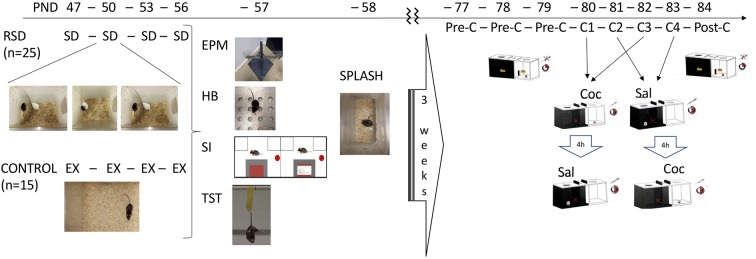
Experimental design. Two groups of mice were used. One group was exposed to repeated social defeat (RSD, *n* = 25). On postnatal day (PND) 47, 50, 53 and 56, experimental mice were introduced into the cage of an aggressive opponent. The physical contact between them was allowed for only 5 min when the experimental mouse experienced social defeat (SD). On the same PND, the other group (CONTROL, *n* = 15) explored (EX) an empty cage. On PND 57, all mice performed the elevated plus maze (EPM) and the hole board (HB), social interaction (SI) and tail suspension test (TST). On PND 58, all mice performed the splash test. After an interval of 3 weeks, all mice underwent the conditioned place preference (CPP) paradigm. On PND 77, 78 and 79, they underwent the pre-conditioning (Pre-C) phase. On PND 80, 81, 82, 83 they performed four conditioning sessions (C1-C4) receiving 1 mg/kg of cocaine (Coc) or saline (Sal) before being placed to the drug- or saline-paired compartment, respectively. On PND 84, mice underwent the post-conditioning (Post-C) phase.

After an adaptation period, the experimental mice (C57BL/6) were assigned to two groups: one non-stressed control group (*n* = 15) and another subsequently exposed to four episodes of RSD (*n* = 25) on PND 47, 50, 53 and 56. On PND 57–58, all mice underwent different behavioral tests: elevated plus maze (EPM), hole board, social interaction, tail suspension, and splash tests. Afterward, all mice were housed in the vivarium for 3 weeks, after which they underwent the CPP procedure (see Figure 1). All experiments took place during the dark period (8.30–16.30) and in a different environment to that of the confrontation sessions. In order to facilitate adaptation, mice were transported to the dimly illuminated experimental room 1 h prior to testing.

### Experimental Protocols

#### Repeated Social Defeat (RSD)

The RSD procedure consisted of four encounters (separated by intervals of 72 h, PND 47, 50, 53 and 56) with a conspecific isolated mouse (OF1), which resulted in the defeat of the experimental animal. Each encounter lasted for 25 min and consisted of three phases, which began by introducing the experimental animal (intruder) into the home cage of the aggressive opponent (resident) for 10 min. During this initial phase, the intruder was protected from attack by a wire mesh wall, which allowed social interaction and threats from the aggressive male resident. The wire mesh was then removed from the cage and the confrontation between the two mice began and lasted for 5 min. In the third phase, the wire mesh was returned to the cage to separate the two animals once again for another 10 min to allow for social threats by the resident. Intruder mice were exposed to a different aggressor mouse during each episode of social defeat. The criterion used to define an animal as defeated was the adoption of a specific posture signifying defeat, characterized by an upright submissive position, limp forepaws, upwardly angled head, and retracted ears (Miczek et al., 1982; Ribeiro Do Couto et al., 2006). All experimental mice displayed defeat, given that they all faced resident mice with high levels of aggression. The first and fourth agonistic encounters were videotaped and evaluated by an observer who was blind to the treatment (Brain et al., 1989) using a computerized system (Raton Time 1.0 software; Fixma SL, Valencia, Spain). The time spent in avoidance/flee and defense/submission by the experimental mice and the time spent in threat and attack by the resident aggressive mice were measured, as were the latencies of these behaviors. The control (non-stressed) group underwent the same protocol, without the presence of a “resident” mouse in the cage (exploration).

#### Elevated Plus Maze (EPM)

The effects of RSD on anxiety were evaluated using the EPM paradigm on PND 57. This test is based on the natural aversion of mice to open elevated areas, as well as on the natural spontaneous exploratory behavior they exhibit in novel environments; therefore, it measures the extent to which rodents avoid high open spaces. The apparatus consisted of two open arms (30 × 5 cm) and two enclosed arms (30 × 5 cm), and the junction of the four arms formed a central platform (5 × 5 cm). The floor of the maze was made of black Plexiglas and the walls of the enclosed arms were made of clear Plexiglas. The open arms had a small edge (0.25 cm) to provide the animals with additional grip. The entire apparatus was elevated 45 cm above floor level. The total time spent in the open and closed arms, the number of entries into the open and closed arms, and the percentage of time and entries into the open arms are commonly considered indicators of open space-induced anxiety in mice. Thus, anxiety levels are considered to be lower when the measurements in the open arms are higher and the measurements in the closed arms are lower, and vice versa (Rodgers and Johnson, 1995; Rodgers and Dalvi, 1997). Moreover, the total entries into the closed arms are regarded as locomotor activity scores (Campos et al., 2013; Valzachi et al., 2013).

At the beginning of each trial, subjects were placed on the central platform facing an open arm and were allowed to explore for 5 min. The maze was cleaned with a 7% alcohol swab after each test, and the device remained untouched until completely dry. The behavior of the mice was video recorded and later analyzed by an investigator blind to the experimental conditions, using a computerized method (Raton Time 1.0 software; Fixma SL, Valencia, Spain). The measures recorded during the test period were frequency of entries and time spent in each section of the apparatus (open arms, closed arms and central platform). An arm was considered to have been visited when the animal placed all four paws on it. The following measures were taken into account for the statistical analyses: the latency to first enter the open arms, the time and percentage of time [(open/open + closed) × 100] spent in the open arms, the number and the percentage of open arm entries and total entries into the arms.

#### Hole Board Test

The novelty-seeking of mice was evaluated in the hole board test 24 h after the last defeat or exploration (PND 57). This test was carried out in a square box (28 × 28 × 20.5 cm) with transparent Plexiglas walls and 16 equidistant holes of 3 cm in diameter on the floor (CIBERTEC SA, Madrid, Spain). Photocells below the surface of the holes detected the number of times that mice performed a head-dip. At the beginning of the test, mice were placed in the same corner of the box and were allowed to freely explore the apparatus for 10 min. The latency to the first dip and the frequency of dips were automatically recorded by the apparatus.

### Social Interaction Test

Twenty-four hours after the last defeat or exploration (PND 57), the social behavior of the mice was evaluated in an open field (37 × 37 × 30 cm). A perforated plexiglass cage (10 × 6.5 × 30 cm) was placed in the middle of one wall of the open field. After habituation to the room, each animal was placed in the center of the open field and was allowed to explore it twice, under two different experimental conditions. The first time (object phase), the perforated plexiglass cage was empty. After 10 min of exploration, the experimental mouse was returned to its home cage for 2 min. Next, a mouse of the OF1 strain was confined to the perforated cage (to safeguard the experimental mouse from attack) and the experimental mouse was reintroduced in the open field for 10 min (social phase). The OF1 mouse was unfamiliar to the experimental mouse (i.e., it was different from the one used in the RSD episodes). In both phases, the time spent in the 8 cm area surrounding the perforated cage—the interaction zone—was registered and automatically sent to a computer using the Ethovision 2.0 software package (Noldus, Wageningen, The Netherlands). An index of social interaction (ISI) was obtained [time spent in the interaction zone during the social phase/(time spent in the interaction zone during the social phase + time spent in the interaction zone during the object phase); Henriques-Alves and Queiroz, 2016]. The ISI is commonly used as the social preference-avoidance index (Krishnan et al., 2007).

#### Tail Suspension Test (TST)

The tail suspension test (TST) measures the behavioral variable of immobility, which is considered to represent despair (Pollak et al., 2010). It is based on the observation that rodents, after initial escape-oriented movements, develop an immobile posture when placed in an inescapable, stressful situation. In the case of the TST, the stressful situation involves the hemodynamic stress of being hung in an uncontrollable fashion by their tail (Cryan et al., 2005). This has been used as a measure of behavioral depression because, when antidepressant treatments are given prior to the test, the subjects engage in escape-directed behaviors for longer periods of time than after treatment with a vehicle (Pollak et al., 2010).

Twenty-four hours after the last defeat or exploration (PND 57), we investigated whether our procedure of social defeat modified the length of time spent in immobile positions in the TST. Following the protocol described by Vaugeois et al. (1997), mice were suspended by the tail, using adhesive tape, from a hook connected to a strain gauge that recorded their movements during a 6-min test period. The behavior displayed by the mice was video recorded and later analyzed by an observer blind to the treatment received by the animal, using a computerized method (Raton Time 1.0 software; Fixma SL, Valencia, Spain). The parameters considered for the statistical analyses were the total time spent immobile and the latency to show immobility.

#### Splash Test

The splash test consisted of spraying a 10% sucrose solution on the dorsal coat of a mouse placed in a transparent cage (15 × 30 × 20 cm) with regular bedding to stimulate grooming behavior. The behavior of the mice was videotaped for 5 min and later analyzed by an observer blind to the treatment received by the animal using a computerized method (Raton Time 1.0 software; Fixma SL, Valencia, Spain). The latency to the first grooming, the time spent engaged in this behavior and its frequency were recorded. An increase in the latency of grooming and a decrease in the time and/or frequency of grooming is interpreted as depressive-like behavior (Smolinsky et al., 2009).

#### Conditioned Place Preference (CPP)

Three weeks after the last episode of social defeat, the animals carried out the CPP procedure. For place conditioning, we employed eight identical Plexiglas boxes with two equal-sized compartments (30.7 cm long × 31.5 cm wide × 34.5 cm high) separated by a gray central area (13.8 cm long × 31.5 cm wide × 34.5 cm high). The compartments had different colored walls (black vs. white) and distinct floor textures (fine grid in the black compartment and wide grid in the white one). Four infrared light beams in each compartment of the box and six in the central area allowed the recording of the position of the animals and their crossings from one compartment to the other. The equipment was controlled by three IBM PC computers using MONPRE 2Z software (Cibertec SA, Madrid, Spain).

The CPP consisted of three phases and took place during the dark cycle following an unbiased procedure in terms of initial spontaneous preference (for detailed explanations of the procedure, see Maldonado et al., 2007). In brief, during pre-conditioning (Pre-C), the time spent by the animal in each compartment during a 15-min period was recorded. Animals showing a strong unconditioned aversion or a preference for a given compartment were excluded from the study. In the second phase (conditioning), which lasted for 4 days, experimental animals received saline before being confined to the vehicle-paired compartment for 30 min and, after an interval of 4 h, were injected with 1 mg/kg of cocaine immediately before being confined to the drug-paired compartment for 30 min. During the third phase, or post-conditioning (Post-C), the time spent by the untreated mice in each compartment was recorded during a 15-min period.

### Statistical Analysis

The effects of RSD on the different behavioral measures (with the exception of CPP) were evaluated by means of unpaired Student *t*-tests, comparing the non-stressed control group to the defeated group (control vs. RSD). In the case of CPP, a mixed two-way ANOVA with a within-subjects variable Days with two levels (Pre-C and Post-C) and a between-subjects variable Stress with two levels (Control and RSD) was used. *Post hoc* comparisons were performed with Bonferroni tests, which allow multiple hypotheses to be tested simultaneously, limiting the type I error rate without increasing the probability of a type II error occurring.

With the data obtained in the defeat episodes and in the behavioral tests performed 24 or 48 h afterward (EPM, hole board, social interaction, tail suspension and splash tests), the group of defeated mice was separated into two subgroups according to the median of the whole group. Mice with scores higher than the median were assigned to the High Score group and those with lower scores to the Low Score group. For example, defeated mice were defined as high or low novelty-seeking (NS) according to their head-dip scores (below or above the defeated group median) in the hole board test. We have previously used this median-split analysis to study the effects of NS on the behavioral effects of different drugs of abuse (Arenas et al., 2014; Montagud-Romero et al., 2014; Mateos-García et al., 2015; Rodríguez-Arias et al., 2015, 2016). A one-way ANOVA with a between-subjects variable—Group, with three levels (Control, Defeated High Score and Defeated Low Score)—was performed for the following measures: time in defense/submission in the first episode of defeat, percentage of time in the open arms of the EPM, number of dips in the hole board, time of immobility in the TST, and grooming (frequency and time) in the splash test. The *post hoc* comparison was performed with the Tukey test. To determine the possible behavioral markers of resilience to the effects of social defeat on cocaine CPP, a mixed two-way ANOVA with a within-subjects variable—Days, with two levels (Pre-C and Post-C)—and a between-subjects variable—Group, with three levels (Control, Defeated High Score and Defeated Low Score)—was used. *Post hoc* comparisons were performed with Bonferroni tests.

In order to determine whether there was a relationship among the performances of mice in the different procedures, Pearson correlation tests were used. In the case of CPP, the conditioning score (time spent in Post-C minus time spent in Pre-C) was calculated. All statistical analyses were performed with the SPSS program.

## Results

### Effects of RSD on the CPP Induced by Cocaine

The ANOVA of the CPP data showed a significant effect of the variable Days (*F*_(1,38)_ = 5.634; *p* < 0.05) and the Interaction Days × Stress (*F*_(1,38)_ = 4.186; *p* < 0.05). RSD increased the rewarding effects of cocaine since only defeated mice spent more time in the drug-paired compartment in Post-C than in Pre-C (*p* < 0.001). Conversely, mice not exposed to defeat (Control group) did not show CPP (Supplementary Figure S1).

### Behavioral Profile of Mice During Social Defeats and Resilience to Cocaine CPP

After the behavioral analysis of defeat episodes, defeated mice were divided into two subgroups according to their Defense/Submission scores during the first episode of defeat (below or above the median of the defeated group, 20.11 s, Low or High Defense/S. Student *t*-test showed a significant difference between these two subgroups of defeated mice (Low and High Defense/S) with respect to the Time spent in Defense/Submission in the first episode of defeat (*t*_(26)_ = −5.878; *p* < 0.001).

**Figure 2:**
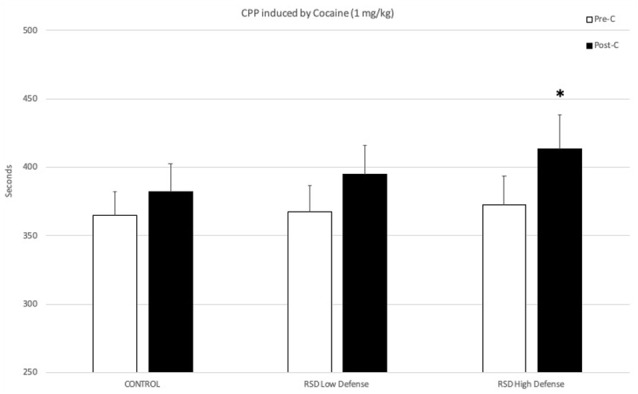
Effects of RSD on cocaine-induced CPP according to the behavioral profile of mice during the first episode of defeat. One group of mice was not exposed to stress (CONTROL, *n* = 15) and the other group was exposed to RSD (*n* = 25). The group of defeated mice was divided into two subgroups according to the time spent in defense/submission in the first episode of defeat: RSD Low Defense and RSD High Defense. After defeat, mice were conditioned with cocaine. Bars represent the mean (±SEM) time (in seconds) spent in the drug-paired compartment in the pre-conditioning (Pre-C, white bars) and the post-conditioning test (Post-C, black bars). *P < 0.05, significant difference in the time spent in the drug-paired compartment in Post-C vs. Pre-C test.

The behavioral profile of mice during the defeat episodes is related to their subsequent resilience or vulnerability to developing cocaine-induced CPP. The ANOVA of the CPP data of the control group and the two groups of defeated mice separated in function of the Time spent in submissive behavior during the first episode of defeat showed that the variable Days (*F*_(1,37)_ = 11.179; *p* < 0.01) and the interaction Days × Group (*F*_(2,37)_ = 3.297; *p* < 0.05) were significant. Bonferroni *post hoc* comparisons revealed that only the High Defense/S group, which spent more time in defensive/submissive behavior, showed CPP (*p* < 0.05, significantly longer time in the drug-paired compartment in Post-C than in Pre-C). The control group (non-defeated mice) and the Low Defense/S group (defeated mice that showed less time in defensive/submissive behaviors) did not develop CPP (see Figure 2).

**Figure 3:**
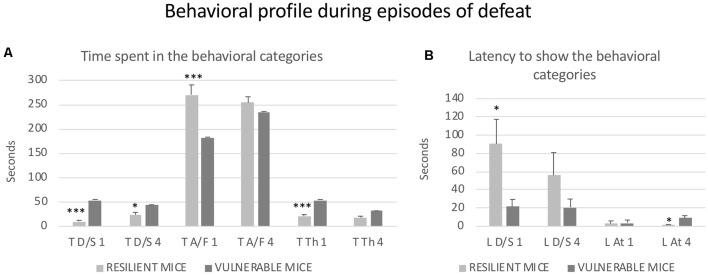
Behavioral profile during episodes of the defeat of mice that were resilient to the long-term effects of RSD on a cocaine-induced CPP. All mice were exposed to RSD (*n* = 25) and later conditioned with cocaine. **(A)** Bars represent the mean (±SEM) time (in seconds) spent in the behavioral categories of defense/submission (T D/S), avoidance/flee (T A/F) and threat (T Th) analyzed during the first and fourth episodes of defeat. **(B)** Bars represent the mean (±SEM) latency (in seconds) to show defense/submission (L D/S) and attack (L At). As can be seen in Figure 2; defeated mice that did not acquire cocaine-induced CPP were defined as resilient mice (light gray bars), while defeated mice that developed cocaine-induced CPP were defined as vulnerable mice (dark gray bars). *P < 0.05, ***P < 0.001, significant difference vs. vulnerable mice.

Besides the Time spent in Defense/Submission, the behavioral analysis of defeat episodes revealed other differences among the mice that were resilient or vulnerable to the effects of RSD on the CPP induced by cocaine. Student’s *t*-tests showed significant differences between the two subgroups of defeated mice in the first episode of defeat with respect to Latency of Submission (*t*_(26)_ = 2.322; *p* < 0.05), Time spent in Flight (*t*_(26)_ = 4.519; *p* < 0.001) and Time receiving Threat from the opponent (*t*_(26)_ = −4.01; *p* < 0.001). Moreover, subgroups of defeated mice showed differences in the fourth episode of defeat in the Time spent in Defense/Submission (*t*_(26)_ = −2.075; *p* < 0.05) and in the Latency of Attack from the opponent (*t*_(26)_ = −2.334; *p* < 0.05). As can be seen in Figure 3, the behavioral profile of resilient mice was characterized by lower submission and more avoidance/flee. In addition, they received lower levels of threat but were attacked faster.

### Elevated Plus Maze and Resilience to Cocaine CPP

**Figure 4:**
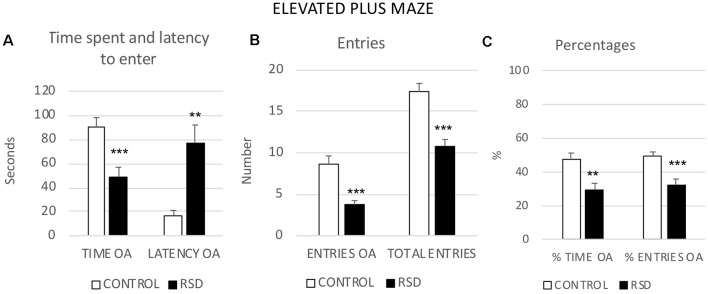
Effects of RSD on the EPM. One group of mice was not exposed to stress (CONTROL, *n* = 15) and the other group was exposed to RSD (*n* = 25). The behavior of mice in the EPM was evaluated. **(A)** Bars represent the mean (±SEM) time (in seconds) spent in the open arms (OA) of the maze and the mean (±SEM) latency to enter to the OA. **(B)** Bars represent the mean (±SEM) number of entries into the arms of the maze. **(C)** Bars represent the mean (±SEM) percentages of time spent in and entries into the OA. **P < 0.01, ***P < 0.001, significant difference with respect to CONTROL group.

RSD induced anxiogenic-like effects in the EPM. Student’s *t*-tests showed significant differences between defeated and control mice in several measures related to the open arms. In comparison to controls, mice exposed to RSD showed a decrease in the Time (*t*_(42)_ = 3.407; *p* < 0.001) and Percentage of time (*t*_(42)_ = 3.143; *p* < 0.01) spent in the open arms, an increase in the latency to enter the open arms (*t*_(40)_ = −3.174; *p* < 0.01), and a reduced number of Entries (*t*_(42)_ = 5.780; *p* < 0.001) and Percentage of entries (*t*_(42)_ = 3.493; *p* < 0.001) into the open arms. Furthermore, RSD decreased the total number of Total entries into the arms (*t*_(42)_ = 5.410; *p* < 0.001; Figure 4).

**Figure 5:**
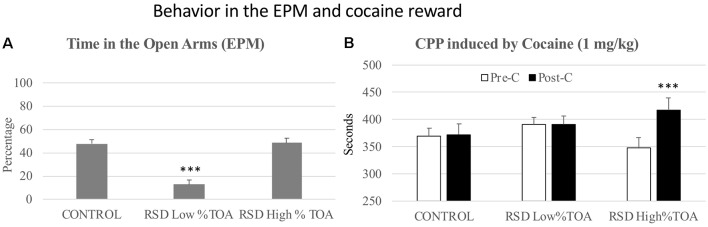
Behavior in the elevated plus-maze and cocaine reward. **(A)** Resilience to the short-term effects of RSD on the EPM. One group of mice was not exposed to stress (CONTROL, *n* = 15) and the other group was exposed to RSD (*n* = 25). The group of defeated mice was divided into two subgroups according to the percentage of time they spent in the open arms (%TOA): RSD Low %TOA and RSD High %TOA. Bars represent the mean (±SEM) percentage of TOA. ***P < 0.001, significant difference vs. the CONTROL and RSD High %TOA groups. **(B)** Effects of RSD on cocaine-induced CPP according to the behavioral profile of mice in the EPM. Mice (CONTROL, RSD Low % TOA and RSD High % TOA groups) were conditioned with cocaine. Bars represent the mean (±SEM) time (in seconds) spent in the drug-paired compartment in the pre-conditioning (Pre-C, white bars) and the post-conditioning test (Post-C, black bars). ***P < 0.001, significant difference in the time spent in the drug-paired compartment in Post-C vs. Pre-C test.

In order to evaluate resilience to the effects of RSD in the EPM, defeated mice were divided into two subgroups according to their scores of Percentage of time in the open arms (below or above the median of the defeated group, 25.92%), and Low or High %TOA. A one-way ANOVA showed a significant effect of the variable Group (*F*_(2,41)_ = 41.326; *p* < 0.001). Tukey *post hoc* comparisons indicated that the Low %TOA group was significantly different from the control and High %TOA groups (*p*s < 0.001; Figure 5A). Thus, there was a group of mice that were resilient to the effects of RSD on the EPM and did not show a decrease in the percentage of time spent in the open arms.

However, resilience to the anxiogenic-like effects of RSD in the EPM is inversely related to resilience to the long-term effects of RSD on cocaine-induced CPP. The ANOVA of the CPP data of controls and the two groups of defeated mice (Low and High %TOA) showed that the variable Days (*F*_(1,38)_ = 8.046; *p* < 0.01) and the Interaction Days × Group (*F*_(2,38)_ = 3.806; *p* < 0.05) were significant. Bonferroni *post hoc* comparisons showed that only the mice that spent the higher percentage of time in the open arms (High %TOA) developed CPP (*p* < 0.001, more time in the drug-paired compartment in Post-C than in Pre-C). The control group (non-defeated mice) and the group of defeated mice that spent the lower percentage of time in the open arms (Low %TOA) did not develop CPP (see Figure 5B).

Besides the percentage of time in the open arms, there were other differences in the open arm measures between mice that were resilient and vulnerable to the long-term effects of RSD on cocaine-induced CPP. Student’s *t*-tests indicated significant differences between both groups of defeated mice with respect to the time spent (*t*_(26)_ = −5.937; *p* < 0.001), number of entries (*t*_(26)_ = −3.341; *p* < 0.01) and percentage of entries into the open arms (*t*_(26)_ = −4.619; *p* < 0.001). It appeared that mice that were resilient to the long-term effects of RSD on cocaine-induced CPP engaged less in the exploration of the open arms (see Supplementary Figure S2).

### Hole Board Test and Resilience to Cocaine CPP

**Figure 6:**
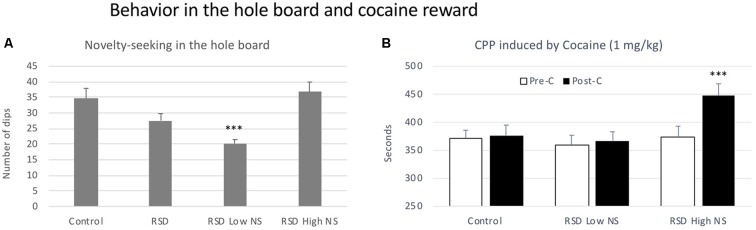
Behavior in the hole board test and cocaine reward. **(A)** Short-term effects of RSD on novelty-seeking behavior in the hole board. One group of mice was not exposed to stress (CONTROL, *n* = 15) and the other group was exposed to RSD (*n* = 25). The behavior of mice in the hole board test was evaluated. The group of defeated mice (RSD, second bar) was divided into two subgroups according to the number of dips they performed: RSD Low NS and RSD High NS. Bars represent the mean (±SEM) number of dips. ***P < 0.001, significant difference vs. the CONTROL and RSD High NS groups. **(B)** Effects of RSD on cocaine-induced CPP according to the novelty-seeking (NS) profile of mice. Mice (CONTROL, RSD Low NS and RSD High NS groups) were conditioned with cocaine. Bars represent the mean (±SEM) time (in seconds) spent in the drug-paired compartment in the pre-conditioning test (Pre-C, white bars) and the post-conditioning test (Post-C, black bars). ***P < 0.001, significant difference in the time spent in the drug-paired compartment in Post-C vs. Pre-C test.

No significant effects of RSD were observed in the latency to the first dip, but defeated mice showed an almost significant reduction in the number of dips (*t*_(40)_ = 1.930, *p* < 0.06; Figure 6A, second bar).

In order to evaluate resilience to the effects of RSD in the hole board test, defeated mice were divided into two subgroups according to their dip scores (below or above the median of the defeated group, 26 dips), Low novelty-seeking (Low NS) or High NS. A one-way ANOVA revealed a significant effect of the variable Group (*F*_(2,39)_ = 12.91, *p* < 0.001). Tukey *post hoc* comcrent from the control group and from the High NS group (*p*s < 0.001; Figure 6A). Thus, this group of mice was resilient to the effects of RSD on the hole board test and did not show a decrease in the number of dips.

Resilience to the effects of RSD in the hole board test is inversely related to resilience to the long-term effects of RSD on cocaine-induced CPP. The ANOVA of the CPP data of the control group and the two groups of defeated mice separated in function of the number of dips (Low and High NS) showed that the variable Days (*F*_(1,38)_ = 9.41, *p* < 0.004) and the Interaction Days × Group (*F*_(2,38)_ = 3.65, *p* < 0.04) were significant. Bonferroni *post hoc* comparisons revealed that only mice in the RSD High NS group developed CPP (*p* < 0.001, more time in the drug-paired compartment in Post-C than in Pre-C). The control group (non-defeated mice) and the group of defeated mice with fewer dips (RSD Low NS) did not develop CPP (see Figure 6B).

### Social Interaction and Resilience to Cocaine CPP

**Figure 7:**
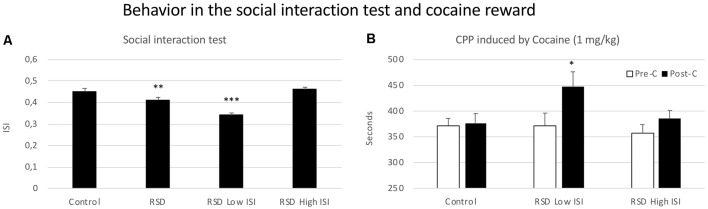
Behavior in the social interaction test and cocaine reward. **(A)** Short-term effects of RSD on the social interaction test. One group of mice was not exposed to stress (CONTROL, *n* = 15) and the other group was exposed to RSD (*n* = 25). The behavior of mice in the social interaction test was evaluated. The group of defeated mice (RSD, second bar) was divided into two subgroups according to their index of social interaction (ISI): RSD Low ISI and RSD High ISI. Bars represent the mean (±SEM) ISI. **P < 0.01, significant difference with respect to the CONTROL group; ***P < 0.001, significant difference with respect to the CONTROL and RSD Low ISI groups. **(B)** Effects of RSD on cocaine-induced CPP according to the behavioral profile of mice in the social interaction test. Mice (CONTROL, RSD Low ISI and RSD High ISI groups) were conditioned with cocaine. Bars represent the mean (±SEM) time (in seconds) spent in the drug-paired compartment in the pre-conditioning test (Pre-C, white bars) and the post-conditioning test (Post-C, black bars). *P < 0.005, significant difference in the time spent in the drug-paired compartment in Post-C vs. Pre-C test.

Mice exposed to RSD showed a reduced ISI when they were exposed to an aggressive OF1 mice (*t*_(39)_ = 2.924; *p* < 0.01; Figure 7A, second bar). However, this reduction was not observed in all the defeated mice. According to their ISI score (below or above the median of the defeated group, 0.43), defeated mice were separated into two groups: Low ISI or High ISI. A one-way ANOVA revealed a significant effect of the variable Group (*F*_(2,39)_ = 42.231, *p* < 0.001). Tukey *post hoc* comparisons indicated that the Low ISI group was significantly different from the control and High ISI groups (*p*s < 0.001; Figure 7A). Thus, there was a group of defeated mice that was resilient to the impairing effects of RSD on social interaction and that did not engage in less social interaction.

The ANOVA of the CPP data of the control group and the two groups of defeated mice separated in the function of their ISI showed that the variable Days (*F*_(1,37)_ = 12.032; *p* < 0.001) and the interaction Days × Group (*F*_(2,37)_ = 3.508; *p* < 0.05) were significant. *Post hoc* comparisons revealed that only the RSD Low ISI group displayed CPP (*p* < 0.05, significantly higher time spent in the drug-paired compartment in Post-C than in Pre-C). The control group of mice not exposed to defeat and the group of defeated mice that showed a higher social interaction index (RSD High SI group) did not develop CPP (see Figure 7B).

### Tail Suspension Test and Resilience to Cocaine CPP

**Figure 8:**
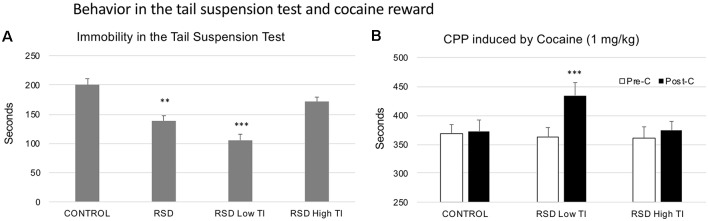
Behavior in the TST and cocaine reward. **(A)** Short-term effects of RSD on the TST. One group of mice was not exposed to stress (CONTROL, *n* = 15) and the other group was exposed to RSD (*n* = 25). The behavior of mice in the TST was evaluated. The group of defeated mice (RSD, second bar) was divided into two subgroups according to the time spent immobile (TI): RSD Low TI and RSD High TI. Bars represent the mean (±SEM) time (in seconds) spent immobile. **P < 0.01, significant difference with respect to CONTROL group; ***P < 0.001, significant difference with respect to the CONTROL and RSD High NS groups. **(B)** Effects of RSD on cocaine-induced CPP according to the behavioral profile of mice in the TST. Mice (CONTROL, RSD Low TI and RSD High TI groups) were conditioned with cocaine. Bars represent the mean (±SEM) time (in seconds) spent in the drug-paired compartment in the pre-conditioning test (Pre-C, white bars) and the post-conditioning test (Post-C, black bars). ***P < 0.001, significant difference in the time spent in the drug-paired compartment in Post-C vs. Pre-C test.

With respect to the control group, RSD reduced the Time spent immobile by the mice (*t*_(42)_ = 4.452; *p* < 0.0; Figure 8A; second bar), but did not affect the Latency to show this behavior (data not shown).

In order to evaluate resilience to the effects of RSD in the TST, defeated mice were divided into two subgroups according to their scores of Time spent immobile (below or above the median of the defeated group, 141 s, Low TI or High TI. One-way ANOVA showed a significant effect of the variable Group (*F*_(2,41)_ = 27.728; *p* < 0.001). Tukey *post hoc* comparisons indicated that the group that spent less time in immobility (Low TI) was significantly different from the control and High TI groups (*p*s < 0.001; Figure 8A). Thus, there was a group of mice that was resilient to the effects of RSD on the TST and that did not show a decrease in immobility.

Resilience to the effects of RSD in the tail suspension is associated with resilience to the long-term effects of RSD on cocaine-induced CPP. The ANOVA of the CPP data of the control group and the two groups of defeated mice separated in function of the Time spent immobile showed that the variable Days (*F*_(1,38)_ = 11.029; *p* < 0.01) and the Interaction Days × Group (*F*_(2,38)_ = 3.320; *p* < 0.05) were significant. Bonferroni *post hoc* comparisons showed that only the Low TI group developed CPP (more time in the drug-paired compartment in Post-C than in Pre-C (*p* < 0.001). The control (non-defeated mice) and the RSD High TI groups did not develop CPP (see Figure 8B).

### Splash Test and Resilience to Cocaine CPP

**Figure 9:**
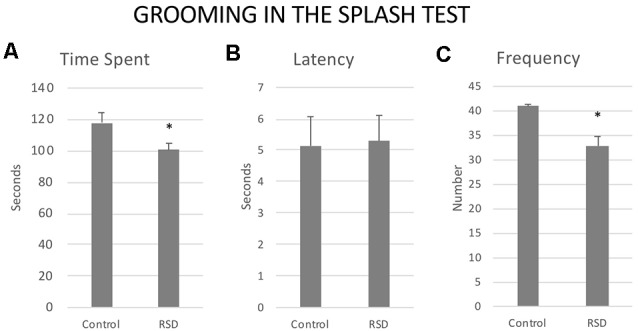
Effects of RSD on grooming behavior in the splash test. One group of mice was not exposed to stress (CONTROL, *n* = 15) and the other group was exposed to RSD (*n* = 25) on PND 47, 50, 53 and 56. The grooming behavior of mice in the splash test was evaluated. **(A)** Bars represent the mean (±SEM) time (in seconds) spent grooming. **(B)** Bars represent the mean (±SEM) latency (in seconds) to groom. **(C)** Bars represent the mean (±SEM) number of times that the mice perform grooming. *P < 0.05, significant difference with respect to Control group.

Exposure to RSD reduced the Frequency (*t*_(40)_ = 2.37; *p* < 0.05) and the Time spent in Grooming (*t*_(40)_ = 2.407; *p* < 0.05). No significant effects were observed with respect to the Latency to the first grooming (*t*_(40)_ = −0.115; *p* < 0.9; Figure 9).

**Figure 10:**
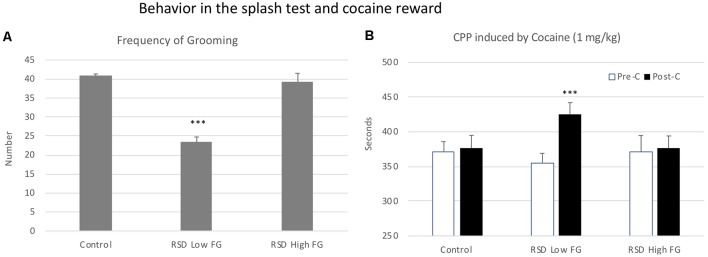
Behavior in the splash test and cocaine reward. **(A)** Resilience to the short-term effects of RSD on grooming behavior in the splash test. One group of mice was not exposed to stress (CONTROL, *n* = 15) and the other group was exposed to RSD (*n* = 25). The grooming behavior of mice in the splash test was evaluated. The group of defeated mice was divided into two subgroups according to their frequency of grooming (FG): RSD Low FG and RSD High FG. Bars represent the mean (±SEM) number of times that the mice performed grooming behavior. ***P < 0.001, significant difference vs. the CONTROL and RSD High FG groups. **(B)** Effects of RSD on cocaine-induced CPP according to the behavioral profile of mice in the splash test. Mice (CONTROL, RSD Low FG and RSD High FG groups) were conditioned with cocaine. Bars represent the mean (±SEM) time (in seconds) spent in the drug-paired compartment in the pre-conditioning test (Pre-C, white bars) and in the post-conditioning test (Post-C, black bars). ***P < 0.001, significant difference in the time spent in the drug-paired compartment in Post-C vs. Pre-C test.

In order to evaluate resilience to the effects of RSD in the splash test, defeated mice were divided into two subgroups according to their scores of Frequency of grooming (below or above the median of the defeated group, 33.8 times), Low FG or High FG. One-way ANOVA showed a significant effect of the variable Group (*F*_(2,39)_ = 15, 758; *p* < 0.001). Tukey *post hoc* comparisons indicated that the group RSD Low FG differed significantly from the control and the RSD High FG groups (*p*s < 0.001; Figure 10A). Thus, this group of mice was resilient to the effects of RSD on the splash test and did not show a decrease in grooming.

Resilience to the effects of RSD in the splash test is associated with resilience to the long-term effects of RSD on cocaine-induced CPP. The ANOVA of the CPP data of the control group and the two groups of defeated mice separated in function of their frequency of grooming showed that the variable Days (*F*_(1,36)_ = 7.82, *p* < 0.01) and the Interaction Days × Group (*F*_(2,36)_ = 3.230; *p* < 0.05) were significant. Bonferroni *post hoc* comparisons showed that only the RSD Low FG group developed CPP (more time in the drug-paired compartment in Post-C than in Pre-C (*p* < 0.001). The control (non-defeated mice) and the RSD High FG groups did not develop CPP (see Figure 10B).

When defeated mice were divided into two subgroups according to their scores in Time spent grooming (below or above the median of the defeated group, 103.22 s), Low TG or High TG, the one-way ANOVA showed a significant effect of the variable Group (*F*_(2,39)_ = 16, 32; *p* < 0.001) and Tukey *post hoc* comparisons indicated that the group RSD Low TG (mean 81.38, SD 3.35) was significantly different from the control (mean 118, SD 6.3) and the RSD High TG (mean 116, SD 4.38) groups (*p*s < 0.001). However, no influence of this behavioral trait on the CPP induced by cocaine was observed. The ANOVA of the CPP data of control, RSD Low TG and RSD High TG showed that only the variable Days was significant (*F*_(1,36)_ = 5.53, *p* < 0.05; data not shown).

### Correlations Between Measurements in the Different Behavioral Tests

A limited number of significant correlations were observed among the performances of mice in the different behavioral procedures (see Supplementary Table S1). There was a correlation between the percentage of time in the open arms in the EPM and the time spent immobile in the TST (*r* = 0.504; *p* < 0.01), as well as a negative correlation between the time spent in submission and the ISI (*r* = −0.403; *p* < 0.05). The CPP score correlated with the number of dips (*r* = 0.421; *p* < 0.05), with mice with a higher novelty-seeking proving to be more vulnerable to developing cocaine-induced CPP. Furthermore, there was a significant inverse correlation between the ISI and the CPP score (*r* = −0.393, *p* < 0.05), since mice with reduced social interaction were more likely to show CPP.

## Discussion

The results of the present work reveal individual differences in the response of mice to RSD exposure during late adolescence. During defeat experiences, some mice displayed less defense/submission and more avoidance/flee behaviors, while others were characterized by the opposite pattern. In the short term, RSD induced anxiety-like symptoms in the EPM, social avoidance in the social interaction test, hyperreactivity in the TST and depressive-like symptoms in the splash test. In the long term, RSD increased the sensitivity of mice to the rewarding effects of a low dose of cocaine in the CPP paradigm. However, only one subgroup of mice showed anxiety- or depression-like symptomatology, a reduction of novelty-seeking, deficits of social interaction, increased reactivity to stress, and greater vulnerability to cocaine-induced CPP (vulnerable mice), while another subgroup remained resilient to the effects of RSD. More importantly, the behavioral profile of the mice in the short-term response to RSD was predictive of subsequent resilience to the long-term influence of RSD on cocaine reward. The defeated mice characterized by lower levels of defensive/submissive behavior, less interest in the open arms in the EPM, less novelty-seeking behavior, a greater level of social interaction, greater immobility in the TST and a higher frequency of grooming in the splash test were resilient to the RSD-induced potentiation of cocaine CPP.

### Resilience to the Long-Term Effects of RSD on Cocaine CPP Is Associated With the Behavioral Profile of Mice During Social Defeat Episodes

After the behavioral analysis of the defeat episodes, defeated mice could be segregated into two subpopulations. In the first episode of defeat, one group (resilient mice) displayed a more active coping response characterized by a longer latency to show submission, less time engaged in defense/submission and more time in avoidance/flee, while the other group (vulnerable mice) showed the opposite behavioral profile. In the fourth episode of defeat, resilient mice showed less defense/submission and were attacked faster by the opponent, which suggests that they managed the stressful situation better than vulnerable mice. The coping response of experimental animals exposed to stress has been used in other studies to distinguish between resilient and vulnerable individuals. For example, male rats were classified as having an active or passive coping strategy according to their latency of submission (Wood et al., 2010, 2013; Pearson-Leary et al., 2017; Grafe et al., 2018; Corbett et al., 2019) and the index of flee behavior in a social interaction test performed after RSD has also been applied to mice (Henriques-Alves and Queiroz, 2016).

Since an active coping strategy has been related with resilience to the negative consequences of stress (Feder et al., 2009; Wu et al., 2013; Wood and Bhatnagar, 2015), we hypothesized that the behavioral profile of mice during defeat episodes is predictive of the long-term effects of RSD on the CPP induced by cocaine. As expected, RSD exposure during late adolescence increased the sensitivity of mice to the rewarding effects of cocaine in adulthood, since only defeated mice developed CPP after conditioning with a dose that was ineffective in non-stressed control mice. These results are in line with and extend our previous findings in OF1 strain mice (Montagud-Romero et al., 2017; Rodríguez-Arias et al., 2017; Ferrer-Pérez et al., 2018; García-Pardo et al., 2019). However, this is the first study to demonstrate that certain mice are resilient to the long-term RSD-induced potentiation of cocaine reward. In a previous study, Krishnan et al. (2007) exposed mice to 10 episodes of social defeat and segregated them (on day 11) into susceptible and unsusceptible subjects according to the presence or absence of social avoidance; in other words, only susceptible mice (showing social avoidance) exhibited cocaine-induced CPP. Whether the effect of defeat on cocaine reward continued to be present long after RSD was not evaluated since both resilient and vulnerable mice performed the CPP procedure 24 h after the last session of defeat (Krishnan et al., 2007). Our results indicate that the behavioral profile of late adolescent mice during episodes of defeat is an early predictor of their subsequent susceptibility or resilience to the effects of RSD on cocaine-induced CPP in adulthood. Vulnerable defeated mice with higher levels of submission developed CPP. Conversely, defeated mice that developed a more active coping strategy during defeat episodes were resilient, as they behaved like control mice and did not acquire CPP. These results are in accordance with those observed by Yanovich et al. (2018), who reported that only selectively bred submissive (but not dominant) mice displayed a marked increase in cocaine CPP after exposure to chronic mild stress. A specific coping strategy is considered to be adaptive (i.e., it reduces the impact of stress on the subject) depending on the environment and the type of stressor (Wood and Bhatnagar, 2015); our results suggest that, in conditions of repeated exposure to brief episodes of social stress, passive coping (such as submissive behaviors and immobility) is less adaptive.

### Resilience to the Long-Term Effects of RSD on Cocaine CPP Is Inversely Related With Resilience to the Anxiety-Like Behavior Induced by RSD in the EPM

Our results show that RSD induces a behavioral profile in the EPM argued to be indicative of anxiety (Campos et al., 2013). In comparison to non-stressed controls, defeated mice spent less time and a lower percentage of time in the open arms of the EPM, performed fewer entries and percentage of entries into these arms, and displayed longer latency to visit an open arm for the first time. These results are in agreement with previous studies reporting that different procedures of social stress induce anxiety-like symptomatology in the EPM (Rodgers and Cole, 1993; Lehmann and Herkenham, 2011; Iñiguez et al., 2014; Duque et al., 2017).

Nevertheless, not all defeated mice showed an aversion for the open arms. Subpopulations could be segregated into those that are susceptible and resilient to the short-term effects of RSD on the EPM. Resilient mice spent a similar percentage of time in the open arms to the control group, which was not exposed to RSD. In contrast, vulnerable mice spent a clearly lower percentage of time in the open arms in comparison to controls and to the other group of defeated mice. Kaufmann and Brennan (2018) also identified a subgroup of defeated mice that spent less time in the open arms (which were also vulnerable to the social avoidance induced by RSD) and another subgroup that was resilient to both deficits. Other studies have also affirmed the existence of animals that are resilient to the effects of several types of social stress on the EPM. For example, using the predator odor stress model, rats were segregated as susceptible or resilient based on EPM behavior and context avoidance (Brodnik et al., 2017). Similarly, in another study, rats were classified as vulnerable or resilient to the effects of RSD on anxiety according to the behavior they displayed in the EPM, dark/lightbox and acoustic startle response test (0 or 1 symptom = resilient rat, 2 or 3 symptoms = vulnerable rat; Le Dorze and Gisquet-Verrier, 2016). In this way, it would seem that some animals are resilient to the anxiety-like behavior induced by social stress.

Due to the close association between anxiety and cocaine use disorders (Vorspan et al., 2015), it can be hypothesized that subjects that are resilient to the effects of RSD on anxiety in the EPM are also resilient to the long-term effects on cocaine reward. However, our results do not support this theory. Unexpectedly, the defeated mice that did not develop cocaine CPP were those that spent a lower percentage of time in the open arms. In contrast, the defeated mice spending a higher percentage of time in the open arms (which were, thus, resilient to the short-term effects of social defeat) showed an enhanced vulnerability to cocaine and developed CPP. No previous studies have evaluated whether the behavioral profile in the EPM after exposure to RSD is related to subsequent vulnerability or resilience to developing cocaine-induced CPP. Krishnan et al. (2007) did not observe a relationship between the expression of anxiety-like symptoms in the EPM and the acquisition of cocaine-induced CPP in mice exposed to RSD. In the study in question, vulnerable mice (which displayed social avoidance and cocaine-induced CPP) and resilient mice (that did not show these effects) exhibited an increase in the time spent in the closed arms in the EPM (Krishnan et al., 2007). Conversely, in a more recent study, rats that were vulnerable to the stress induced by exposure to the odor of a predator were more sensitive to the effects of cocaine (Brodnik et al., 2017). Seven days after stress exposure, male rats were segregated into resilient or susceptible groups according to the time they spent in the open arms of the EPM and in the compartment associated with the predator’s odor. In comparison to resilient rats, the hyperactivity induced by cocaine and the reinforcing effect of this drug in the self-administration paradigm were enhanced in susceptible rats (Brodnik et al., 2017). These divergent results may be due to differences in the methodology (species, type of stress, the time elapsed between stress exposure and behavioral testing, etc.). However, from our point of view, the most important factor is the criterion used to discriminate resilient animals from vulnerable animals. In the study by Brodnik et al. (2017), rats were considered vulnerable when they met both criteria: less than 50 s in the open arms and less than 20 s in the odor-associated compartment. In this way, it can be assumed that rats showing an anxiety/fear response to stress are more vulnerable to the effects of cocaine. Conversely, in the present study, mice that spent a lower percentage of time in the open arms of the EPM were resilient to the long-term effects of RSD and did not develop cocaine-induced CPP. It is not logical to assume that the mice with higher anxiety levels were less vulnerable to cocaine; thus, we propose other interpretations of the results obtained. The EPM test not only reveals an anxious state but might also suggest behavioral disinhibition. In this sense, the longer time spent in the open arms by vulnerable mice that developed CPP might indicate a pre-existing impulsive phenotype (Gass et al., 2014) that predisposes them to be more vulnerable to the effects of cocaine. Furthermore, it is important to consider that the EPM entails a conflict between two natural tendencies: the motivation to stay in the protected closed arms, naturally associated with safety, and the motivation to explore the non-protected open arms, which may be associated with a potential danger or a threat (Ennaceur and Chazot, 2016). There is no objective evidence as to the real significance of a reduction in the open arms measures: i.e., whether it represents anxiety or a sense of security. From our point of view, the mice that were resilient to the long-term effects of RSD on cocaine reward were those that, after experiencing an attack from an opponent, actively avoided the open arms to stay safe from other potential threats.

### Resilience to the Long-Term Effects of RSD on Cocaine CPP Is Related With the Novelty-Seeking Profile of Defeated Mice in the Hole Board Test

Exposure to RSD induced a reduction in the number of dips in the hole board test in some defeated mice, since a tendency to such reduction was observed only in the group of defeated mice as a whole (*n* = 25, *p* = 0.06 with respect to controls). In rodents, novelty-seeking behavior has been defined as a “preference for” or a tendency to increase the exploration of novel objects and environments (Nadal-Alemany, 2008; Belin et al., 2011; Vidal-Infer et al., 2012). A very limited number of studies have evaluated the influence of stress exposure on novelty-seeking behavior, and the few data reported are controversial. In male rats, RSD did not modify their behavior in the hole board test 24 h after the last defeat (Albonetti and Farabollini, 1994), but chronic RSD reduced directed exploration in mice (Erhardt et al., 2009). Conversely, rats chronically exposed to predator odor before and during puberty showed increased novelty-seeking during late adolescence (Toledo-Rodriguez and Sandi, 2011). Such discrepant results are probably due to the different developmental periods in which the animals were exposed to stress. From our point of view, the lower number of dips in the hole board test in the subgroup of defeated mice could have been due to the fact that RSD induced an emotional arousing state that motivated a reduced exploration of a novel, potentially dangerous environment.

The influence that the novelty-seeking trait exerts on vulnerability to stress and drug use has been repeatedly demonstrated (Kabbaj et al., 2001; Duclot et al., 2011; Vidal-Infer et al., 2012; Duclot and Kabbaj, 2013; Clinton et al., 2014; Hodges et al., 2018). In particular, novelty-seeking behavior is one of the personality factors that may explain individual differences in vulnerability to drug abuse (Dellu et al., 1996). Higher novelty-seeking has been identified as a risk factor for the initiation of drug use and transition to abuse (Kelley et al., 2004; Staiger et al., 2007; Milivojevic et al., 2012; Mateos-García et al., 2015). In line with this idea, we observed that the subgroup of mice showing greater novelty-seeking after RSD was more vulnerable to the rewarding effects of cocaine. Conversely, mice performing a significantly lower number of dips (that is, mice that responded to RSD with emotionality or avoidance of a novel environment) remained resilient to the long-term effects of RSD on cocaine reward and did not develop CPP. These results, together with those observed in the EPM, lead us to assume that defeated mice that avoid potential risk are protected from the subsequent consequences of social stress on the rewarding effects of cocaine.

### Resilience to the Long-Term Effects of RSD on Cocaine CPP Is Associated With Resilience to the Social Avoidance Induced by RSD in the Social Interaction Test

Exposure to RSD produced a short-term deficit of social interaction. This reduction of the ISI in defeated mice has been associated with the social avoidance that characterizes affective disorders (Golden et al., 2011), and has been repeatedly observed after RSD or social instability (Krishnan et al., 2007; Golden et al., 2011; Henriques-Alves and Queiroz, 2016; Browne et al., 2018; Dong et al., 2018; Hodges et al., 2018). Furthermore, the ISI is the most used measure to distinguish between mice that are resilient or vulnerable to the effects of different models of social defeat (Krishnan et al., 2007; Chaudhury et al., 2013; Donahue et al., 2014; Friedman et al., 2014; Hodes et al., 2014; Isingrini et al., 2016; Sun et al., 2016; Nelson et al., 2018; Prabhu et al., 2018; Gururajan et al., 2019). In this line, we have also observed a subgroup of resilient mice (with similar ISI to that of control mice) and another subgroup of vulnerable mice that displayed social avoidance. It must be taken into account that the type of opponent used in the social interaction test has an influence on the results observed. In the present study, the use of the OF1 strain instead of the strain employed as experimental animals probably induced a more pronounced social avoidance in defeated mice. In fact, it has been reported that, when the target in the social interaction test was a C57BL/6J mouse, both susceptible and resilient mice spent more time in the interaction zone than when the opponent was an aggressive CD1 mouse (Han et al., 2014). Notwithstanding, even when the opponent was of the same strain, the social interaction was significantly higher in resilient than in susceptible mice (Han et al., 2014).

Defeated mice resilient to social avoidance were also resilient to the long-term effects of RSD on cocaine reward. Only the subgroup of defeated mice with a deficit of social interaction developed CPP after conditioning with a low dose of cocaine that was ineffective in non-stressed control mice and in resilient defeated mice. Similar results have been observed by Krishnan et al. (2007), who reported that only mice with a deficit of social interaction (susceptible) developed CPP after conditioning with 5 mg/kg of cocaine 24 h after social defeat, while unsusceptible mice without social avoidance did not develop CPP. Similarly, vulnerable mice with lower levels of social interaction showed reduced alcohol self-administration in comparison to control mice not exposed to stress and to resilient animals without a social interaction deficit (Nelson et al., 2018).

### Resilience to the Long-Term Effects of RSD on Cocaine CPP Is Associated With the Resilience to Hyperreactivity Induced by RSD in the Tail Suspension Test

Exposure to RSD reduced the amount of time spent immobile in the TST, an unexpected result taking into account that immobility in this test has been considered to be depression-like behavior (Katz, 1982; Cryan et al., 2005; Pollak et al., 2010). However, other studies have shown that stressed mice spent less time being immobile than control mice in the tail suspension (Brockhurst et al., 2015) or in the forced swim tests (Suo et al., 2013; Sadler and Bailey, 2016). In contrast, other researchers have reported that RSD did not affect immobility 24 h after the last episode of defeat (Kinsey et al., 2007; Krishnan et al., 2007), or even increased it in defeated mice identified as vulnerable in a social interaction test (Dong et al., 2017). As Commons et al. (2017) stated, the behavioral alterations observed in the TST must be interpreted with caution, since this paradigm may model the stress-coping strategy from which depressive-like behavior is inferred. Besides, the use of the TST can be problematic in the case of C57BL/6 mice, as they have a propensity to climb using their tails (Can et al., 2012). In the present study, the decrease in immobility in defeated mice could be attributed to inoculation against stress; however, we suspect that such an effect is related to an enhanced reactivity of defeated mice to the situation of moderate inescapable stress that the TST represents. In contraposition to the conventional interpretation of immobility in the forced swim and TSTs as behavioral despair (Katz, 1982), it has been understood by some to represent enhanced anxiety (van Dijken et al., 1992). In support of this idea, a subgroup of defeated mice exhibiting less immobility in the TST reduced their consumption of sucrose, a behavior associated with the lack of interest in pleasurable activities that characterizes depression (Bowens et al., 2012). In the same line, we observed that RSD decreased the frequency of grooming in the splash test, an effect interpreted as depressive-like symptomatology (see the following section). Considered together, these results suggest that the decreased immobility of defeated mice in the TST should be interpreted as an enhanced reactivity to this stressful situation, rather than a reduction of depressive-like behavior.

In addition, our results indicate that vulnerable mice that are more immobile in the TST are more sensitive to the rewarding effects of cocaine and CPP acquired with a low dose of this drug. Conversely, resilient mice with immobility values similar to controls and not exposed to stress did not develop CPP. Thus, mice that were resilient to RSD-induced hyperreactivity were also resilient to the long-term effects of RSD on cocaine reward.

### Resilience to the Long-Term Effects of RSD on Cocaine CPP Is Associated With Resilience to Depressive-Like Behavior Induced by RSD in the Splash Test

Exposure to RSD decreased the duration and frequency of grooming in the splash test, considered a relevant measure of the motivational state of animals (Butelman et al., 2019). A reduction of grooming behavior has been observed after exposure to different stressors (Jolles et al., 1979; Spruijt et al., 1992; Charney, 2004; Smolinsky et al., 2009; Heaney et al., 2011; Veloso et al., 2016; Szewczyk et al., 2019), and has been interpreted as anhedonia, as it is reversed by antidepressant drugs (Brachman et al., 2016; de Souza et al., 2019).

In addition, we have observed that some defeated mice remained resilient to the depressive-like behavior induced by RSD. Although there are no studies with the splash test, Krishnan et al. (2007) demonstrated that mice that were resilient to the effects of RSD in the social interaction test were also resilient to depression-like behavior evaluated with the sucrose preference test. Conversely, a recent study that segregated mice into resilient and vulnerable subjects according to their immobility values in the TST showed that vulnerable mice with higher immobility spent more time engaged in grooming and exhibited this behavior more frequently in an unfamiliar cage (Reis-Silva et al., 2019). A possible explanation for these divergent results is the different type of stressor used (RSD vs. tail suspension) and the controversial interpretation of the results obtained in the TST (as commented before, greater immobility has been interpreted as depression-like behavior and as lower reactivity to moderate inescapable stress). In the present study, the resilience to the short-term effects of RSD on the frequency of grooming predicted subsequent resilience to cocaine reward; only vulnerable mice with reduced grooming behavior acquired cocaine-induced CPP 3 weeks after RSD. Similar results were reported by Krishnan et al. (2007) 1 day after the last episode of defeat, as only mice with anhedonia (indicated by a lower sucrose preference) showed cocaine CPP.

### Correlation Between Behavioral Markers of Resilience to the Long-Term Effects of RSD on the CPP Induced by Cocaine

As discussed in previous sections, the segregation of experimental animals into vulnerable or resilient subpopulations with respect to the effects of stress on cocaine reward has been the subject of only two studies. Brodnik et al. (2017) observed that the reinforcing efficacy of cocaine in the self-administration paradigm was lower in mice that were resilient to the effects of stress (predator odor exposure) on EPM behavior and context avoidance. Previously, Krishnan et al. (2007) had reported that mice resilient to the effects of RSD on social interaction were also resilient to developing anhedonia and cocaine-induced CPP a short time after defeat. They also observed that the resilient phenotype regarding social interaction (but not regarding depressive-like behavior) persisted 4 weeks after defeat; however, the potential long-term enhanced vulnerability to the rewarding effects of cocaine was not evaluated (Krishnan et al., 2007). The results of the present work are in accordance with and extend those obtained in the aforementioned studies. Our main contribution is to demonstrate that some behavioral profiles of the short-term response to social stress predict the subsequent resilience of defeated mice to the rewarding effects of cocaine. Resilient mice that did not develop cocaine CPP were less submissive during defeat episodes, a behavioral profile associated with an active coping with stress (Finnell et al., 2017; Pearson-Leary et al., 2017; Grafe et al., 2018), which in turn is associated with resilience to developing mental disorders. Furthermore, resilient mice avoided the open arms of the EPM and showed less novelty-seeking in the hole board test, which can be interpreted as active avoidance-risk behavior (in concordance with the higher avoidance/flee behavior observed during the first defeat episode). Mice resilient to developing cocaine CPP were also resilient to social avoidance in the social interaction test, hyperreactivity in the TST and depressive-like behavior in the splash test.

We have attempted to establish a potential association of the different resilient phenotypes by means of correlations between the variables shown to be indicative of resilience to the long-term effects of RSD on cocaine reward (lower defense/submission, lower percentage of time in open arms, lower novelty-seeking, higher ISI, higher immobility in the TST and higher frequency of grooming). Furthermore, the contribution of each individual variable to cocaine resilience was determined by correlating these variables with the CPP score. There was a correlation between the time spent in submission and the ISI: the mice that showed less submission during the defeat episodes were resilient to developing a deficit of social interaction. The percentage of time in the open arms of the EPM correlated with the time spent immobile in the TST; thus, the behavior in both tests seemed to be associated in some way. In light of these results, we hypothesize that mice that are less reactive to stress (i.e., those that show more immobility) feel less of a need for safety in the EPM. The number of dips negatively correlated (although non-significantly, *p* < 0.073) with the frequency of grooming, which may indicate that mice that respond to social defeat with lower novelty-seeking are also more resilient to developing anhedonia. With respect to the CPP scores, only two correlations were statistically significant. First, the correlation between CPP score and the number of dips indicated that the novelty-seeking profile was a strong predictor of resilience or vulnerability to the rewarding effects of cocaine. Second, the correlation between CPP score and ISI indicated that social avoidance induced by RSD was associated with enhanced vulnerability to the rewarding effects of cocaine. These correlations suggest that resilience to the effects of social defeat on cocaine reward may be a result of particular behavioral traits or the combination of several behavioral traits. An important fact is that most of the behavioral tests used in the present study measure unrelated behaviors. However, even in the absence of a correlation with the CPP score, the response of defeated mice in each one of these behavioral tests was predictive of its subsequent resilience or vulnerability to cocaine reward. The main relevance of these results is that they show that cocaine use disorders should be considered from a multi-dimensional perspective. Such disorders result from the interaction of biological and behavioral processes that are altered by environmental factors, such as stress exposure. Some individual behavioral traits, such as the level of novelty-seeking or the degree of social interaction, may confer, by themselves, an enhanced or reduced responsivity to cocaine reward. However, more frequently, a complex neurobehavioral profile resulting from the combination of two or more behavioral traits contributes in a cumulative way to resilience or vulnerability to developing a drug addiction.

## Conclusion

In the present study, we demonstrate that resilience to the long-term potentiation of the rewarding effects of cocaine-induced RSD is associated with different behavioral profiles. Resilient mice are characterized by less submission during defeat episodes, less interest in the open arms in the EPM, lower novelty-seeking, less reactivity in the TST, and an absence of RSD-induced deficits such as social avoidance and anhedonia (see Table 1). A limitation of the present work is the use of the median to discriminate between vulnerable and resilient mice in the behavioral procedures. With this approach, we defined as resilient any mouse below or above the median depending on the test and variable used. However, it is certainly improbable that 50% of the subjects were constantly resilient to the different effects of social defeat stress. In future studies, we will employ larger samples of defeated mice and quartiles (rather than the median) to divide them into resilient and non-resilient subjects, in order to give a more substantiality to the notion of resilience.

**Table 1:** Summary table of results.

Behavioral test		Resilient mice	Vulnerable mice
Conditioned place preference	Cocaine CPP	=	↑
Agonistic encounters	Defense/Submission	↓	↑
Elevated plus-maze	Open Arms Measures	↓	=
Hole board test	Novelty-seeking	↓	=
Social interaction test	Social investigation	=	↓
Tail suspension test	Immobility	=	↓
Splash test	Grooming	=	↓

*After RSD exposure, vulnerable mice developed CPP with a low dose of cocaine, that did not induce CPP in controls and defeated resilient mice. Reduced defensive/submissive behavior during episodes of defeat, avoidance of the open arms of the elevated plus-maze and lower novelty-seeking were behavioral traits predictive of resilience to the effects of RSD on cocaine CPP. Defeated resilient mice behaved as controls in the social interaction, tail suspension and splash test. Conversely, increased defensive/submissive behavior during episodes of defeat, hyperreactivity in a stressful situation (tail suspension test) and depressive-like behaviors (social avoidance and anhedonia after RSD) were behavioral traits predictive of vulnerability to the effects of RSD on cocaine CPP*.

The general conclusion of this study, based on the data from all the tests performed, is that several individual traits, including an active coping response, and avoidance of potential dangers in unknown environments, and reduced acute stress reactivity, contribute to a subject’s resilience to the negative consequences of social stress (deficit of social interaction, anhedonia and enhanced drug sensitivity). From a translational point of view, our results support the real-world observation that not all individuals exposed to social stress during late adolescence subsequently suffer from mental disorders. For example, not all adolescents exposed to bullying develop cocaine use disorders in adulthood. Resilient subjects have less probability of showing symptoms of post-traumatic stress disorder after a traumatic event (Tugade and Fredrickson, 2004; Wrenn et al., 2011; Lee et al., 2014), while more vulnerable subjects can suffer from mental disorders and addictive behaviors in response to this level of stress. In this context, it is necessary to promote in vulnerable individuals attitudes and personality traits that are characteristic of resilience. According to our results, and to evidence in humans, an active coping strategy (Feder et al., 2009) and a search for social support (Wu et al., 2013) should be encouraged. Individuals with an active coping response attempt to change their perception of the stressful stimulus (Wu et al., 2013) by means of cognitive reevaluation, which may increase positive thinking, another individual factor associated with resilience (Meredith et al., 2011; Holz et al., 2019). In addition, it is necessary to decrease reactivity to stressful events and increase awareness of dangers, as well as to promote the self-control function and sense of safety. These can be achieved by means of problem-solving tasks, relaxation training and cognitive restructuration (Thompson et al., 2018).

Future works should address ways to increase resilience in vulnerable animals. The negative consequences of stress can be reduced through environmental manipulations (Greenwood and Fleshner, 2008; Schloesser et al., 2010; MacKay et al., 2017) and by allowing mastication during stress exposure, a model of active behavioral coping in rodents (Hennessy and Foy, 1987; Hori et al., 2004; Kubo et al., 2009; Stalnaker et al., 2009; Helmreich et al., 2012). Finally, it is important to study the neurobiological substrates of resilience, which underly the behavioral phenotypes observed in our study. There are recent reviews about the causes of resilience that highlight the importance of neuroplasticity in several brain networks, changes at the blood-brain barrier, genetic factors, and the role of the immune system, the metabolism and the gut microbiota (Cathomas et al., 2019; Feder et al., 2019; Holz et al., 2019; Tsyglakova et al., 2019; Turkson et al., 2019). Based on previous studies in our laboratory, we propose that a reduced inflammatory response, epigenetic changes (lower histone acetylation activity), reduced permeability of the BBB, and lower glutamate activity in the brain reward system may mediate the phenotype of resilience to the effects of RSD on cocaine reward (Montagud-Romero et al., 2016a, 2017; Rodríguez-Arias et al., 2017; García-Pardo et al., 2019). Understanding the individual traits and the neurobiological mechanisms that promote resilience may give rise to multiple new approaches to prevention and the development of pharmacological or behavioral interventions that can increase resilience to the negative sequelae of stress and their influence on drug addiction and other mental disorders.

## Data Availability Statement

The raw data supporting the conclusions of this article will be made available by the authors, without undue reservation, to any qualified researcher.

## Ethics Statement

The animal study was reviewed and approved by Ethics Committee in Experimental Research (Experimentation and Animal Welfare) of the University of Valencia (A1507028485045).

## Author Contributions

MA and MG-P contributed to the conception and design of the study. MA, CC-L, MM-C, and AS-O performed the experiments, organized the databases and performed the statistical analyses. CC-L, MM-C, and AS-O wrote sections of the manuscript. MG-P wrote the complete first draft of the manuscript. MA wrote the final version of the manuscript. All authors contributed to manuscript revision, read and approved the submitted version.

## Conflict of Interest

The authors declare that the research was conducted in the absence of any commercial or financial relationships that could be construed as a potential conflict of interest.

## Abbreviations

CPP: conditioned place preference
EPM: elevated plus maze
FG: frequency of grooming
ISI: index of social interaction
NS: novelty-seeking
PND: post-natal day
RSD: repeated social defeat
TI: time of immobility
TST: tail suspension test
%TOA: percentage of time in open arms.
