# Supplementary material for: Behavioral Traits Associated With Resilience to the Effects of Repeated Social Defeat on Cocaine-Induced Conditioned Place Preference in Mice
Source: Front Behav Neurosci. 2020 Jan 9;13:278. doi: 10.3389/fnbeh.2019.00278 (PMC6962131; doi:10.3389/fnbeh.2019.00278)
Supplement: Supplementary file 1 [file Data_Sheet_1.docx]

Supplementary Material

# Supplementary Figures and Tables

## Supplementary Figures

**Supplementary Figure 1.** Effects of repeated social defeat (RSD) on cocaine-induced CPP. One group of mice was not exposed to stress (CONTROL) and the other group was exposed to four episodes of repeated social defeat (RSD) on PND 47, 50, 53 and 56. Three weeks after the last episode of defeat, all mice were conditioned with 1 mg/kg of cocaine. Bars represent the mean (± SEM) time (in seconds) spent in the drug- paired compartment before conditioning sessions in the pre-conditioning test (Pre-C, white bars) and after conditioning sessions in the post-conditioning test (Post-C, black bars). Only the group of mice exposed to RSD developed CPP (***P < 0.001, significant difference in the time spent in the drug-paired compartment in Post-C versus Pre-C test).

**Supplementary Figure 2.** Behavioral profile of mice resilient to the long-term effects of repeated social defeat (RSD) on cocaine-induced CPP in the elevated plus maze (EPM). All mice were exposed to four episodes of repeated social defeat (RSD, n=25) on PND 47, 50, 53 and 56 and their behavior was analyzed in the EPM 24 hours after the last episode of defeat. Three weeks after they were conditioned with 1 mg/kg of cocaine in the CPP paradigm. a) Bars represent the mean (± SEM) time (in seconds) spent in the open arms (OA). b) Bars represent the mean (± SEM) number of entries into the OA. c) Bars represent the mean (± SEM) percentage of time spent in OA and the mean (± SEM) percentage of entries into the OA. As can be seen in Fig. 5b, defeated mice that did not acquire cocaine-induced CPP (Low %TOA) can be defined as resilient mice (light gray bars), while defeated mice that develop cocaine-induced CPP (Low %TOA) can be defined as vulnerable mice (dark gray bars). In comparison to vulnerable mice, resilient mice showed a reduction of all measures related with OA of the EPM (**P < 0.01, *** P < 0.001, significant difference versus vulnerable mice).

Supplementary Table 1

|  |  | TOA | Dips | CPP | ISI | Groom | Immob | Submis |
| --- | --- | --- | --- | --- | --- | --- | --- | --- |
| TOA | Pearson | 1 | 0,185 | 0,302 | 0,27 | 0,096 | **0,504** | -0,173 |
|  | Significance |  | 0,375 | 0,142 | 0,192 | 0,648 | 0,01 | 0,409 |
|  | N | 25 | 25 | 25 | 25 | 25 | 25 | 25 |
| Dips | Pearson | 0,185 | 1 | **0,421** | -0,323 | -0,365 | 0,05 | -0,001 |
|  | Significance | 0,375 |  | 0,036 | 0,116 | 0,073 | 0,814 | 0,996 |
|  | N | 25 | 25 | 25 | 25 | 25 | 25 | 25 |
| CPP | Pearson | 0,302 | **0,421** | 1 | **-0,393** | 0,006 | 0,013 | -0,014 |
|  | Significance | 0,142 | 0,036 |  | 0,05 | 0,977 | 0,951 | 0,947 |
|  | N | 25 | 25 | 25 | 25 | 25 | 25 | 25 |
| ISI | Pearson | 0,27 | -0,323 | **-0,393** | 1 | 0,096 | 0,027 | **-0,403** |
|  | Significance | 0,192 | 0,116 | 0,05 |  | 0,647 | 0,899 | 0,046 |
|  | N | 25 | 25 | 25 | 25 | 25 | 25 | 25 |
| Groom | Pearson | 0,096 | -0,365 | 0,006 | 0,096 | 1 | 0,163 | 0,148 |
|  | Significance | 0,648 | 0,073 | 0,977 | 0,647 |  | 0,436 | 0,48 |
|  | N | 25 | 25 | 25 | 25 | 25 | 25 | 25 |
| Immob | Pearson | **0,504** | 0,05 | 0,013 | 0,027 | 0,163 | 1 | 0,182 |
|  | Significance | 0,01 | 0,814 | 0,951 | 0,899 | 0,436 |  | 0,384 |
|  | N | 25 | 25 | 25 | 25 | 25 | 25 | 25 |
| Submis | Pearson | -0,173 | -0,001 | -0,014 | **-0,403** | 0,148 | 0,182 | 1 |
|  | Significance | 0,409 | 0,996 | 0,947 | 0,046 | 0,48 | 0,384 |  |
|  | N | 25 | 25 | 25 | 25 | 25 | 25 | 25 |

**Supplementary Table 1.** Pearson correlations between measurements in the different behavioral tests. TOA: Percentage of time in the open arms of the Elevated plus maze. Dips: Number of dips in the Hole board test. CPP: Conditioned place preference score (Time spent in the drug-paired compartment in the Post-C minus the Pre-C). ISI: Index of social interaction in the Social interaction test. Groom: Frequency of grooming behavior in the Splash test. Immob: Time spent in immobility in the Tail suspension test. Submis: Time spent in submission in the first episode of social defeat. Bold values indicate statistical significance.
